# Large Language Models in German Continuing Medical Education Assessments: Protocol for a Fully Crossed Experimental Study

**DOI:** 10.2196/91675

**Published:** 2026-07-28

**Authors:** Leyla Özmen, Christian Burisch, Daniel Gödde, Frank Breuckmann, Jan Ehlers, Timur Sellmann

**Affiliations:** 1 Faculty of Health, Witten/Herdecke University, Alfred-Herrhausen-Straße 50, Witten, North Rhine-Westphalia, 58455, Germany, 49 23029260; 2 Leibniz-Gymnasium Essen, District Government Düsseldorf, Essen, North-Rhine-Westphalia, Germany; 3 Chair of Didactics and Educational Research in Healthcare, Witten/Herdecke University, Witten, North-Rhine-Westphalia, Germany; 4 Department of Pathology and Molecular Pathology, HELIOS University Hospital Wuppertal, University Witten/Herdecke, Witten, North-Rhine-Westphalia, Germany; 5 Department of Cardiology, Pneumology, Neurology and Intensive Care Medicine, Klinik Kitzinger Land, Kitzingen, Bayern, Germany; 6 Department of Cardiology and Vascular Medicine, West German Heart and Vascular Center Essen, University Duisburg-Essen, Essen, North-Rhine-Westphalia, Germany; 7 Department of Anaesthesiology I, Witten/Herdecke University, Witten, North-Rhine-Westphalia, Germany; 8 Department of Anesthesiology and Intensive Care Medicine, Evangelisches Krankenhaus BETHESDA zu Duisburg, Duisburg, Germany

**Keywords:** continuing medical education, CME, large language models, LLMs, clinical competence assessment, educational measurement, artificial intelligence, AI, medical education, Germany

## Abstract

**Background:**

Continuing medical education (CME) is a legal and ethical obligation for physicians in Germany. The rapid rise of large language models (LLMs) such as ChatGPT, Gemini, Claude, and Grok raises concerns about the integrity of CME assessments, as LLMs can already pass German CME tests.

**Objective:**

This study aims to determine whether the choice of document format (searchable PDF, protected PDF, raster PDF, or vector PDF) and LLM influences the ability of LLMs to solve CME test questions at rates exceeding the passing threshold specified for each CME module (typically 70%).

**Methods:**

In a fully crossed within-subjects repeated-measures design, 18 expired CME articles from 3 major German publishers across 6 specialties will be converted into 3 cheating-impeding PDF formats and processed alongside the original PDF files by 4 current LLMs (GPT-5, Claude Sonnet 4, Grok-4, and Gemini 3). This results in 16 model-format combinations. Each model will answer every article 3 times per file-format condition, with outcomes derived from aggregated run-level results. The primary outcome is the proportion of correctly answered questions; the secondary outcome is the pass/fail rate.

**Results:**

The study has been approved by the Witten/Herdecke University Ethics Committee (S-260/2025; dated August 10, 2025) and is preregistered at the Open Science Framework. The study is supported by internal departmental resources only, and no external funding was received. Because this protocol evaluates LLMs using expired CME materials, no human participants are being recruited. Data collection is planned to begin in June 2026 and is expected to last approximately 4 weeks. At the time of manuscript submission, no data have been collected or analyzed. Results are expected to be available after the completion of data collection and statistical analysis in 2026. The analyses will quantify performance differences across document formats; these findings may inform the feasibility of nonsearchable document formats as a temporary measure to reduce LLM-enabled cheating risks in CME contexts.

**Conclusions:**

By quantifying how document format constrains LLM performance, this study aims to evaluate simple technical safeguards that may reduce artificial intelligence–assisted manipulation of CME tests and inform regulators and CME providers about how to balance assessment validity, accessibility, and responsible LLM integration into postgraduate medical education.

## Introduction

Large language models (LLMs) such as ChatGPT and Gemini represent transformative advances in natural language processing, demonstrating near-human proficiency across complex reasoning and knowledge-based tasks [1,2]. Recent studies have shown that ChatGPT achieves passing scores in medical board and licensing examinations, including the United States Medical Licensing Examination, often outperforming medical students [1-13].

In Germany, continuing medical education (CME) is a mandatory component of the professional life of physicians. According to the Federal Medical Association (Bundesärztekammer), licensed physicians must collect 250 CME points within 5 years, primarily through certified educational activities, including reading peer-reviewed CME articles and answering associated test questions [14,15].

However, recent research revealed that nonmedical individuals using GPT-4 could successfully pass CME assessments [5], highlighting vulnerabilities in CME evaluation systems. These findings raise ethical and methodological concerns, as CME credits might no longer reflect genuine physician learning or competence.

Building upon these observations, the present experiment evaluates whether the file format of CME materials impacts LLM performance. Specifically, it tests whether nonsearchable or graphically encoded PDFs (raster or vector) can serve as practical countermeasures to prevent LLM-assisted manipulation of CME assessments [16].

The aim of this study is to determine whether the file format of CME materials (searchable, protected, raster, or vector PDF) modulates the ability of current-generation LLMs (GPT-5, Claude Sonnet 4, Grok-4, and Gemini 3) to correctly answer the associated multiple-choice questions at rates exceeding the 70% passing threshold typically required for CME credit. The null hypothesis states that technical measures have no impact on an LLM’s ability to solve the CME tests. The alternative hypothesis posits that these measures impair this ability.

## Methods

### Study Design

This study uses a fully crossed within-subjects repeated-measures design across 4 file-format conditions and 4 LLMs. Each CME article serves as its own control across file formats, yielding a within-item repeated-measures structure.

To account for the nondeterministic nature of LLMs, each model-format-article combination will be run 3 times, with primary and secondary outcomes being generated from recorded responses to CME test questions.

### Ethical Considerations

The study follows the Declaration of Helsinki and Good Clinical Practice principles. Although no human participants are involved, the study adheres to these ethical principles to ensure transparency, scientific integrity, and responsible AI evaluation. Ethics approval was granted by the Witten/Herdecke University Ethics Committee (S-260/2025; dated October 8, 2025). The study is prospectively registered with the Open Science Framework (OSF; OSF.IO/V96R5).

### Study Material

The sampling frame will consist of expired CME modules made available by the 3 prespecified major German medical publishers included in this study (Deutscher Ärzteverlag GmbH, Springer Medizin, and Georg Thieme Verlag). Eligible modules must contain (1) the complete CME article text, (2) the associated multiple-choice questions, and (3) the official answer key required for scoring. Only expired modules will be included to ensure that CME credit can no longer be claimed retrospectively. Modules that are still active, incomplete, duplicated, or technically unsuitable for standardized file-format conversion will be excluded.

To ensure structured coverage of major clinical domains, the eligible modules will be grouped according to 6 prespecified specialties: internal medicine, surgery, pediatrics, gynecology, neurology, and anesthesiology. Article selection will then be performed using a computer-generated randomization procedure from the eligible pool, with balanced allocation across specialties and publisher sources. This approach is intended to provide a heterogeneous multicenter sample from major German CME providers for a methodological comparison of file-format effects.

The aim of this sampling strategy is not to establish formal statistical representativeness of all German CME materials in Germany but rather to assemble a transparent, reproducible, and clinically diverse sample from major national CME providers under standardized experimental conditions.

All CME articles and the associated multiple-choice questions used in this study are protected by German copyright law (Urheberrechtsgesetz) and remain the intellectual property of the respective publishers (Deutscher Ärzteverlag GmbH, Springer Medizin, and Georg Thieme Verlag). Prior to study initiation, all 3 publishers were formally contacted; were informed in writing about the nature, scope, and purpose of the study; and provided the requested expired CME modules together with explicit permission for their use in this research project. No specific conditions were imposed by the publishers. The corresponding correspondence is on file with the study team and is available upon reasonable request.

Only expired CME modules are included, that is, modules for which CME credit can no longer be claimed retrospectively. This ensures that the study cannot influence the active CME credit market, does not interfere with the publishers’ ongoing commercial interests, and excludes any possibility that LLM-generated answers could be misused to obtain valid CME credits. No original article text, question stems, distractors, or answer keys are reproduced in the manuscript, the supplementary materials, or the OSF repository. CME modules are identified only by anonymized internal codes together with their specialty and publication year range, and quantitative performance data are reported in aggregated form. All technically necessary reproductions generated for the file-format conversions (protected, raster, and vector PDFs) are used exclusively as input for the LLMs under evaluation. The CME materials are entered into the 4 LLMs (GPT-5, Claude Sonnet 4, Grok-4, and Gemini 3) via their official consumer web chat interfaces, in accordance with the CME providers’ terms of service for noncommercial research use. Where available, the interfaces are configured to disable use of submitted content for model training to prevent leakage of copyrighted material into future model versions.

### Technical Implementation of File Formats

Four document types will be generated for each CME article to enable within-article comparisons of file-format effects on LLM performance:

1. Searchable PDF: text-based PDFs in which characters are digitally encoded and machine-readable (PDF files as provided by the publishers)
2. Protected PDF: password-protected PDFs in which printing, modifying the content, and extracting text, images, and other elements are disabled (using 256-bit AES encryption)
3. Raster PDF: rasterized image-based PDFs. These consist of pixels arranged in a fixed grid, similar to digital photographs. Each pixel has a fixed color and position, and when zoomed in, the image becomes blurry due to the absence of additional information.
4. Vector PDF: PDFs that represent content using mathematical paths and Bézier curves. Vector graphics remain crisp and scalable, as shapes are dynamically recalculated rather than composed of static pixels.

### Protected PDF Generation and Verification

Protected PDFs will be generated by applying standardized security settings to the original publisher-provided files using 256-bit AES encryption. These settings will uniformly restrict content modification, printing, and extraction of text, images, and metadata across all documents. The applied protection parameters (including encryption level, permission flags, and software toolchain; eg, Adobe Acrobat [Adobe Inc] or equivalent) will be documented in detail in the appendix or OSF materials to ensure transparency and reproducibility.

The aim of the protected condition is not to alter the visual representation of the document but to assess whether access restrictions alone affect LLM performance despite the unchanged underlying content. Therefore, each protected PDF will undergo a verification step prior to model evaluation. This will include manual inspection to confirm that visual fidelity matches the source document and to verify that text extraction is effectively restricted or blocked under standard conditions. Successful protection will be defined as maintaining full human readability while preventing or substantially limiting direct programmatic access to the textual content.

### Raster PDF Generation and Verification

Raster PDFs will be generated using fixed rendering settings that are standardized across all articles, including prespecified image resolution and compression parameters, to ensure consistent image quality across the study. These settings will be documented in detail in the appendix or OSF materials to enable reproducibility.

The aim of the raster condition is not to create artificially unreadable files but to generate nonsearchable image-based PDFs that remain visually legible for human readers while limiting direct machine-readable text access. Therefore, each rasterized PDF will undergo a quality control check before model evaluation to confirm that page content remains readable and that rendering quality is not degraded to a level at which trivial optical character recognition (OCR) failure would be expected purely because of inadequate technical conversion settings.

### Vector PDF Generation and Verification

In the vector PDF condition, the goal is to preserve a “vector-encoded” visual representation while minimizing direct text extractability. To achieve this, all textual content will be converted into vector outlines (ie, text-to-path conversion) so that no selectable text layer remains. Vector PDFs will be generated using a standardized toolchain (eg, Adobe Acrobat Preflight and/or Ghostscript [Artifex Software] or Inkscape [Inkscape Project]). To verify the intended reduction in extractability, each generated vector PDF will undergo a quality control step using automated text-extraction checks (eg, pdftotext [Glyph & Cog LLC] or equivalent); successful conversion will be defined as yielding no meaningful extracted text (eg, empty output or a negligible character count) while maintaining legibility comparable to the source document. These definitions are crucial, as LLMs differ in their ability to parse and extract text from graphical encodings. While raster formats may impede OCR and tokenization, vector formats retain structured information that might still be exploitable by advanced multimodal models [16-18].

### Participants and Models

The study does not involve human subjects as participants, only 1 “operator” (LÖ). Instead, the 4 “participating” LLMs (ChatGPT [version 5; OpenAI], Claude Sonnet [version 4; Anthropic PBC], Gemini [version 3; Google LLC], and Grok [version 4; xAI]) are treated as experimental agents. Each model will attempt to answer CME questions under 4 distinct format conditions. Responses will be collected and scored according to the official CME answer keys provided by the publishers.

### Reproducibility

To ensure reproducibility of the LLM experiments, we will provide the exact prompt templates used either verbatim in an appendix or via a permanent OSF appendix link. In brief, the prompt will include standardized task instructions, presentation of the current CME material in the assigned file-format condition, and instructions to answer the associated multiple-choice questions exclusively on the basis of the provided material. Core prompt components will be harmonized across models as far as technically feasible. Thus, the purpose of standardization is not to claim empirically proven prompt invariance across models but to minimize and transparently document prompt-related variation as consistently as possible across all study conditions.

Each model-format-article combination will be executed in 3 independent runs to account for stochastic variability in LLM outputs. These repeated runs will be used to derive the binary pass or fail outcome, as defined below. The primary analysis will use the mean accuracy across runs.

### Experimental Conditions

There are 16 model-format combinations, comprising a 4 (file format)×4 (LLM) fully crossed design. The four experimental conditions correspond to the 4 file-format types used in the within-article crossover design:

1. Condition 1 (searchable PDF condition): articles are presented as text-based PDFs accessible to all LLMs, as provided by the publishers.
2. Condition 2 (protected PDF condition): articles are presented as password-protected PDFs with disabled printing, extraction, and content modification.
3. Condition 3 (raster PDF condition): articles are provided as rasterized image-based PDFs, limiting direct text recognition.
4. Condition 4 (vector PDF condition): articles are rendered as vector PDFs, preserving structural outlines but reducing text extractability.

### Validity

The validity of the outcome measure depends critically on the LLMs’ ability to correctly parse and interpret the presented CME materials. Different PDF formats introduce systematic variation in this process: searchable PDFs provide a clean, tokenized text structure that can be processed directly, whereas rasterized PDFs require OCR, which is known to introduce transcription errors, reduce token accuracy, and increase noise in model inputs [2]. To reduce the risk that observed performance losses merely reflect avoidable artifacts of excessively poor rasterization quality, raster PDFs will be generated using fixed standardized rendering settings and subjected to a prespecified quality control step before evaluation. Vector-encoded PDFs may further fragment text into glyphs or graphic paths, increasing parsing difficulty and occasionally exceeding tokenization limits in multimodal models [17-19]. These format-dependent processing differences constitute a potential threat to construct validity, as performance may reflect a model’s visual parsing capability rather than its medical reasoning competence. Therefore, evaluating file-format effects is necessary to isolate true diagnostic performance from artifacts introduced by differential input accessibility.

### Randomization

#### Overview

**Figure 1.**
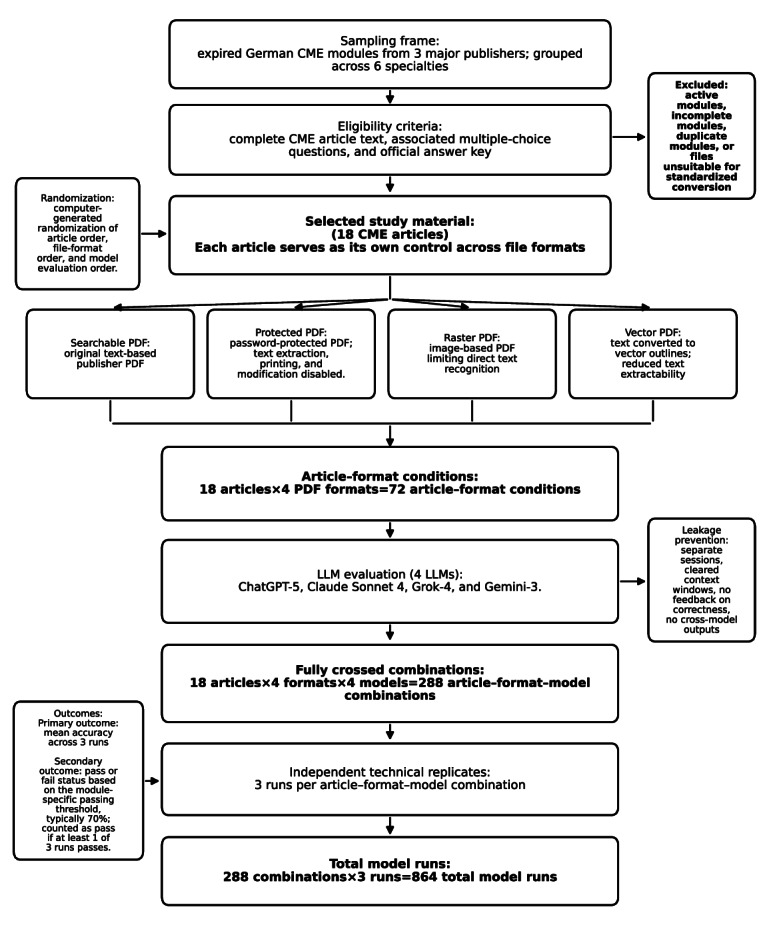
CONSORT (Consolidated Standards of Reporting Trials)–style study flow diagram. CME: continuing medical education; LLM: large language model.

Randomization will occur on three levels: (1) the order in which articles are presented within each model-format sequence, (2) the allocation and order of file formats per article and model, and (3) the order of model evaluation. All randomization sequences will be computer-generated (R version 4.5.0; R Foundation for Statistical Computing; randomization blocks of equal size) and stored in a preregistered allocation file accessible only to the study statistician (see Figure 1 for the CONSORT (Consolidated Standards of Reporting Trials)–style flow diagram of article allocation, randomization, and model evaluation).

The 3 repeated runs for each model-format-article combination will be conducted as independent technical replicates in separate sessions with cleared context windows. They are intended to quantify stochastic output variability rather than to introduce an additional experimental factor. Run order will follow the randomized article-format-model sequence; no additional randomization of replicate order will be performed.

The study flow diagram in Figure 1 illustrates the fully crossed within-subjects repeated-measures design with 18 CME articles, 4 PDF formats, and 4 LLMs, yielding 288 article-format-model combinations. Each combination will be executed in 3 independent runs, resulting in 864 total model runs.

#### Randomization of CME Articles

Each of the 18 CME articles will be converted into all 3 cheating-impeding file formats (protected, raster, and vector), in addition to the original files provided by the publishers. For every article, a randomization list will specify the order in which the 4 formats are presented to each model. This yields a fully crossed within-article repeated-measures structure in which every model processes every article in each file-format condition in a triplicate manner (18 articles×4 formats×4 models×3 runs). Article order within each model-format sequence will be randomized using permuted blocks stratified by clinical specialty to maintain balance across internal medicine, surgery, pediatrics, gynecology, neurology, and anesthesiology.

#### Randomization of File-Format Exposure

For each model and each CME article, the sequence of file formats (searchable, protected, raster, and vector) will be independently randomized using all 24 possible permutations. This ensures that no single format systematically benefits from warm-up or fatigue effects.

#### Randomization of Model Evaluation Order

For each batch of articles, a unique model-order permutation (eg, GPT-5 to Gemini 3 to Claude Sonnet 4 to Grok-4) will be generated.

### Prevention of Cross-Model Leakage

Models will never receive outputs, intermediate reasoning, or corrected answers from prior models. All responses are generated in isolated sessions with cleared context windows. No system prompts contain summaries or previous model outputs. In addition, within-model learning across file formats is minimized by enforcing strict session isolation: the same model never encounters more than 1 format of a given article within a single session, and no feedback on response correctness is provided between runs. Because all prompts start from an empty context window and contain only the current article in a single format, the risk of cumulative learning about individual CME items across formats is substantially reduced.

### Primary Outcome

The primary outcome is the proportion of correctly answered CME questions for each LLM across the 4 file-format conditions (searchable, protected, rasterized, and vector-encoded PDFs). Accuracy will be calculated as the percentage of items answered correctly relative to the official answer key provided by the publishers. For inferential analysis, the 3 repeated runs for each article-model-format combination will be treated as technical replicates and averaged before statistical testing. Thus, the primary inferential unit will be the article-level mean accuracy for each model and file-format condition, rather than the individual run. This approach accounts for stochastic run-to-run variability while avoiding artificial inflation of the effective sample size.

### Secondary Outcome

Pass/fail status will be determined using the passing threshold specified by the respective CME module/provider (typically 70%). The threshold for each module will be recorded from the module instructions. For the binary pass/fail outcome, each model-format-article combination will be classified as “pass” if at least 1 of the 3 runs meets the passing threshold; otherwise, it will be classified as “fail.” This classification takes into account the models’ fundamental ability to pass a CME test despite the measures in effect. For inferential analysis, pass or fail status will therefore be evaluated at the article-model-format level rather than at the individual-run level.

### Statistical Analysis

#### Sample Size and Power Calculation

The primary aim of this study is to quantify the effect of document format on LLM accuracy (searchable, protected, raster, and vector PDFs) within a fully crossed 4 (format)×4 (LLM) repeated-measures design, in which each article serves as its own control across formats. The study includes 18 CME articles, each evaluated under all 16 model-format combinations, with 3 repeated technical runs per article-model-format combination.

The repeated runs will not be treated as independent inferential observations but will be aggregated before hypothesis testing. For the primary outcome, the inferential unit is the CME article within each LLM and file-format condition. Therefore, each predefined comparison of an intervention format against the searchable PDF condition will be based on 18 paired article-level observations per LLM. The sample size calculation was performed for the hypothesis that the use of LLMs would improve CME test results from the guessing probability (20% correct answers, SD σ=40%) to the passing level (70% correct answers) with a confidence level of 1–α=0.95 (ie, α=.05) and a high statistical power of 1*–*β>0.95.

Because CME courses can be completed multiple times without changes to their content or assessment questions, we used a paired design in which randomly selected tests were administered identically across all 16 experimental conditions. To provide a more conservative and clinically diverse sample, we included 18 CME articles. We reviewed the sample size for the binary pass or fail approach of the secondary outcome, again with a confidence level of 1*–*α=0.95 (ie, α=.05) and a high statistical power of 1*–*β>0.95.

A passing probability of 100% was observed in a previous study when an LLM was provided with the complete CME material, which will be the case in this study. Because newer LLM generations are expected to perform at least as well as the models evaluated in the previous study, we conservatively assumed an 80% passing probability under full access to the CME material. If the cheating-impeding measures are effective, performance is expected to drop to a binomial probability of approximately 0.086% for achieving ≥70% correct answers by chance. To remain conservative, we assumed a 20% passing probability under effective cheating-impeding conditions for the sample size calculation.

Under these very conservative settings, we calculated a sample size of 7 CME modules. We again chose a conservative approach and included all 18 CME modules from the previous study.

The final sample size is considered sufficient for detecting substantial, practically meaningful file-format effects while acknowledging that the study is not designed to detect small higher-order interaction effects.

#### Statistical Testing

Descriptive statistics will summarize accuracy and pass/fail rates across LLMs and file-format conditions. Individual run-level results will be retained for descriptive reporting of stochastic variability, but they will not be treated as independent inferential observations.

For the primary outcome, the proportion of correctly answered CME questions will first be calculated for each run. The 3 runs for each article-model-format combination will then be averaged. For each LLM and each file-format condition, the inferential dataset will therefore consist of 18 article-level mean accuracy values.

The primary comparisons will be performed separately for each LLM. Within each LLM, the 3 cheating-impeding file formats—protected PDF, raster PDF, and vector PDF—will each be compared pairwise with the searchable PDF condition. Because the same CME articles are evaluated under all file-format conditions, comparisons will be performed as paired within-article analyses. For each article, the difference in mean accuracy between the intervention format and the searchable PDF condition will be calculated. Depending on the distribution of these paired differences, paired *t* tests or Wilcoxon signed-rank tests will be used. Given the small sample size of 18 articles per LLM, Wilcoxon signed-rank tests will be preferred if normality of the paired differences is not supported.

For the secondary binary pass/fail outcome, pass/fail status will be determined for each article-model-format combination according to the prespecified module-specific passing threshold. For each LLM and file-format condition, the number and percentage of passed CME modules among the 18 articles will be reported. Each intervention format will then be compared with the searchable PDF condition within the same LLM using paired binary analyses. Because the same CME articles are evaluated under both file-format conditions, McNemar-type tests will be used. Given the small number of articles, exact binomial tests based on discordant pairs will be preferred.

Multiplicity adjustment will be applied to the 3 predefined comparisons against the searchable PDF condition within each LLM. Holm adjustment will be used as the primary multiplicity correction. Adjusted *P* values <.05 will be considered statistically significant. All analyses will be performed in R.

## Results

The study has been approved by the Witten/Herdecke University Ethics Committee (S-260/2025; dated August 10, 2025) and is preregistered at the Open Science Framework. The study is supported by internal departmental resources only, and no external funding was received. Because this protocol evaluates LLMs using expired CME materials, no human participants are being recruited. Data collection is planned to begin in July 2026 and is expected to last approximately 4 weeks. At the time of manuscript submission, no data have been collected or analyzed. Results are expected to be available after the completion of data collection and statistical analysis in 2026. The analyses will quantify performance differences across document formats (searchable, protected, rasterized, and vector-encoded PDFs); these findings may inform the feasibility of nonsearchable document formats as a temporary measure to reduce LLM-enabled cheating risks in CME contexts.

## Discussion

### Principal Results

This protocol describes a study designed to test the hypothesis that searchable PDF formats will enable the highest LLM performance on German CME assessments, whereas protected, rasterized, and vector-encoded PDFs will reduce performance by limiting direct machine readability. We further expect that the magnitude of this format effect may vary across model families, depending on their multimodal input-processing capabilities and robustness to OCR-related or layout-related degradation.

If confirmed, these findings would suggest that document format is not a neutral technical property but a potentially relevant determinant of AI solvability in postgraduate medical assessment environments. In this sense, the study is expected to contribute not only to the evaluation of LLM performance in medical education but also to the broader question of whether simple file-format modifications could function as low-threshold safeguards against AI-assisted misuse in CME settings.

### Limitations

This study has several methodological limitations that must be acknowledged. First, LLMs are inherently nondeterministic. Even with fixed prompts and identical input materials, models may produce slightly different outputs across runs due to stochastic sampling, internal state variation, or backend optimization routines.

The study does not include a human control group, preventing direct comparison of how physicians perform with protected, rasterized, or vector-based PDFs compared to LLMs. The experimental setup uses standardized prompts and does not examine the impact of advanced prompt engineering or multistep attacks (eg, external OCR followed by LLM processing), which could potentially circumvent format-based restrictions in real-world cheating scenarios. Although the conflict between AI protection and accessibility for visually impaired users is discussed, it is not empirically investigated.

Finally, modern LLMs are subject to frequent backend updates and performance drift over time, even when model names remain unchanged. As a result, the behavior of the evaluated models at the time of data collection (planned for June 2026) may differ from their performance at the time of publication or future replication attempts. We will therefore document all model version identifiers and run time stamps in detail, but residual version drift remains an inherent limitation of AI evaluation studies.

### Comparison With Prior Work

If protected, rasterized, or vector-encoded CME materials can be shown to reduce LLM performance, such formats may constitute a simple technical safeguard for protecting the integrity of CME assessments by limiting machine readability. However, implementing deliberately nonsearchable formats introduces a significant accessibility conflict. Under the German Accessible Information Technology Ordinance (Barrierefreie-Informationstechnik-Verordnung) and other technical guidelines [20-22], educational materials provided by professional bodies should, where feasible, remain accessible to users with visual impairments, age-related reading limitations, or reliance on assistive technologies such as screen readers or text-to-speech systems. Nonsearchable or access-restricted PDF formats may impair these functions by restricting text extraction, semantic navigation, adjustable contrast, and integrated search tools. Consequently, any attempt to constrain LLM capabilities through file-format manipulation must be balanced against the legal and ethical obligation to ensure accessibility and equal participation for human learners. Future studies should explore hybrid countermeasures that combine technical barriers with authentication protocols (eg, CAPTCHA-style test verification). Beyond regulatory implications, this research contributes to broader discussions on AI governance and digital literacy in postgraduate medical education [20,23-27].

### Future Directions

Future studies should determine whether any observed file-format effects remain stable across newer model generations, other medical specialties, and additional assessment settings beyond CME article-based testing. It will also be important to investigate whether format-based restrictions can be circumvented through combined workflows, such as external OCR, multimodal preprocessing, or prompt-engineered extraction strategies.

In addition, future research should evaluate human-centered alternatives that preserve accessibility while maintaining assessment integrity, including secure authentication procedures, adaptive item delivery, hybrid anticheating measures, and accessibility-preserving technical safeguards. Comparative studies involving human participants may further clarify whether format manipulations disproportionately affect legitimate learners, particularly those who rely on assistive technologies.

### Dissemination Plan

The findings of this study will be disseminated through submission to a peer-reviewed journal and presentation at scientific meetings in the fields of medical education, digital health, and health professions assessment. In addition, the results will be communicated to relevant CME stakeholders, including publishers, professional bodies, and regulatory audiences, to inform ongoing discussions about assessment integrity, accessibility, and responsible AI governance in postgraduate medical education. Where appropriate, study materials relevant to reproducibility will be made available via the OSF in accordance with journal and copyright requirements.

### Conclusions

Rather than merely describing a technical comparison of PDF types, this protocol addresses a timely governance question in postgraduate medical education: whether assessment materials can be made less vulnerable to AI-assisted misuse without creating unacceptable barriers for human users. By examining how searchable, protected, rasterized, and vector-encoded formats affect LLM performance, the study is expected to generate evidence relevant to the design of CME assessments at the intersection of validity, accessibility, and responsible AI integration. The findings may help inform future policy and technical decisions by CME providers, publishers, and regulators.

## Abbreviations

AI: artificial intelligence
CME: continuing medical education
CONSORT: Consolidated Standards of Reporting Trials
LLM: large language model
OCR: optical character recognition
OSF: Open Science Framework
